# Increasing efficiency by optimizing table position for elective primary THA and TKA: a prospective monocentric pilot study

**DOI:** 10.1186/s42836-020-00048-2

**Published:** 2020-10-19

**Authors:** Dirk Zajonz, Celina Höhn, Juliane Neumann, Christine Angrick, Robert Möbius, Gerald Huschak, Thomas Neumuth, Mohamed Ghanem, Andreas Roth

**Affiliations:** 1 Department of Orthopaedic Surgery, Traumatology and Plastic Surgery, University Hospital Leipzig, Liebigstrasse 20, D-04103 Leipzig, Germany; 2 ZESBO – Center for Research on Musculoskeletal Systems, University of Leipzig, Semmelweisstrasse 14, D-04103 Leipzig, Germany; 3 Clinic of Orthopaedic Surgery, Traumatology and Reconstructive Surgery, Zeisigwald Clinics Bethanien Chemnitz, Zeisigwaldstrasse 101, D-09130 Chemnitz, Germany; 4 Innovation Center Computer Assisted Surgery (ICCAS), Leipzig University, Semmelweisstraße 14, D-04103 Leipzig, Germany; 5 Clinic for Anesthesiology and Intensive Therapy, University Hospital Leipzig, Liebigstrasse 20, D-04103 Leipzig, Germany

**Keywords:** Table position, THA, TKA, Increase of efficiency, Operating room

## Abstract

**Introduction:**

Hip and knee arthroplasties are very frequently performed surgeries with high quality standards and continuous optimization potential. Intraoperative processes can be standardized and simplified by optimization of table setups in the operating room to improve the quality and to increase efficiency.

**Patients and methods:**

The existing surgical setups for primary hip and knee arthroplasties in a university maximum care hospital with endoprosthesis center were simulated and analysed with a computer program and optimized setup suggestions were worked out, based on handover times, walking distance and ergonomic aspects determined in the program. In a prospective monocentric analysis, primary hip arthroplasties and knee arthroplasties were examined in currently used and in the new optimized setups (standard procedure according to in-house SOP, senior and main surgeons, no assistants). The surgeries were externally and independently supervised and analysed, whereby the time between incision and suture beginning, handovers per minute and handover times were documented, amongst other things. In addition, an evaluation sheet, which showed the satisfaction with the new setup, was filled by the surgical team.

**Results:**

In the period from April 2016 to December 2018, 19 hip arthroplasties in currently used and 15 in the new optimized setup as well as 9 knee arthroplasties in currently used and 13 in the new setup were performed**.** Attention was paid to constant conditions in the compared groups and disruptive factors (assisted surgeries, complex surgeries, different cementings, etc.) were excluded. In the group of hip arthroplasties, the handover times were significantly different (old 1.82 +/− 1.43 s.; new 1.08 +/− 0.78 s.; *p* <0.001), as well as the handovers per minute (old 1.62 +/− 0.45 handovers/min.; new 2.10 +/− 0.32 handovers/min.; *p* = 0,001). The time between incision and suture beginning indicated no significant difference (old 53.89 +/− 18.92 min.; new 49.73 +/− 12.18 min; *p* = 0.466): During the knee arthroplasties, handovers per minute were significantly different (old 1.83 +/− 0.38 handovers/min.; new 2.40 +/− 0.35 handovers/min.; *p* = 0.002). The time between incision and suture beginning (old 71.11 +/− 20.72 min.; new 70.69 +/− 17.12 min.; *p* = 0.959) and the handover times (old 1.06 +/− 0.64 s.; new 0.91 +/− 0.59 s.; *p* = 0.152) indicated no significant difference. The evaluation of the questionnaires showed a significant difference (*p* < 0.001) in the group of hip arthroplasties in the category “visibility”. For the knee arthroplasties, all items except “visibility” (*p* = 0.261) differed significantly. Overall, a high level of staff satisfaction with the new setup was achieved.

**Conclusions:**

In both groups, more handovers per minute could be achieved in the optimized setup and in the group of the hip arthroplasties, the handover times were significantly faster. The evaluation sheet showed a high satisfaction of the surgical staff with the new setup. No reduction of the time between incision and suture beginning could be determined. This can be attributed to a certain training effect, the adjustment to the setup modification and the low number of cases. The new setup offers a practical alternative for hip arthroplasties as well as for knee arthroplasties as it optimizes the events in the operating room in many ways. For example, there were more handovers per minute possible and passing of the surgical instruments free from interferences. Moreover, it increases the efficiency and achieves a high satisfaction of the staff.

## Introduction

The optimization of processes in hospitals and especially in the operating room holds great potential for increasing efficiency and working more economically [1–4]. The increased demands in the medical service sector with high costs, limited budgets and scarce resources in the healthcare system justify the necessity to constantly increase the efficiency [2]. The operating room represents an important junction in the clinical routine which offers various optimization possibilities. The operating rooms are 10–30% of the whole hospital costs and they are one of the most expensive functional areas in hospitals [5]. With these requirements and processes, the operating room has a central position in the clinical routine, and essential resources in the hospital and the efficiency of the other wards often are dependent on the schedule of the operating room [3]. Seim and Sandberg focused, among other things, on increasing the profitability of operating rooms and the economic optimization possibilities in the clinical routine. A key aspect of their publication, beside reducing delays and increasing the efficiency of anaesthesia, was the increase of the throughput in the operating room, by which a resource optimization with cost saving was achieved [4].

Hip and knee arthroplasties are frequently performed surgeries, which therefore have a high optimization potential. According to the Federal Statistical Office of Germany, endoprosthesis surgeries are among the 20 most frequent surgeries in total [6]. In intraoperative process optimization, the aim is both to rationalize resources and to achieve improvements in quality. An instrument for quality assurance for hip and knee arthroplasties in Germany is certification as an endoprosthesis center. This is initiated by specialist companies which aim to increase patient safety and care quality [7]. The DGOOC (German Association for Orthopaedics and Orthopaedic Surgery), together with the AE (German Society for Endoprosthetics) and the Professional Association of Specialists for Orthopaedics and Trauma Surgery (BVOU) has developed an initiative for the certification of medical organizations for joint replacement, which is based on the proven use of quality-improving treatment elements in the endoprosthetic care of large joints [8]. Elements of the certification include an interdisciplinary organized treatment, a verifiable standard of education and further training for all involved occupational groups and the consistent, at least annual, inspection of observance of the standards by external auditors [7].

An important factor is the structuring of processes for standardization and simplification, to improve the quality and finally to reduce the handover and surgery times. After an analysis of the handover times and the table positions, a new setup was designed using computer simulation [9]. This was integrated and tested in the clinical routine.

The aim of the publication was to optimize the table position in the operating room for primary hip and knee arthroplasties and thereby increase efficiency and staff satisfaction. Both ergonomic and temporal factors of the adjusted table position were assessed in order to be able to optimize more. The question to answer is, if we can reduce the handover and surgery times and thus achieve a high staff satisfaction with the new OR setup in the real clinical routine.

## Materials and methods

**Fig. 1:**
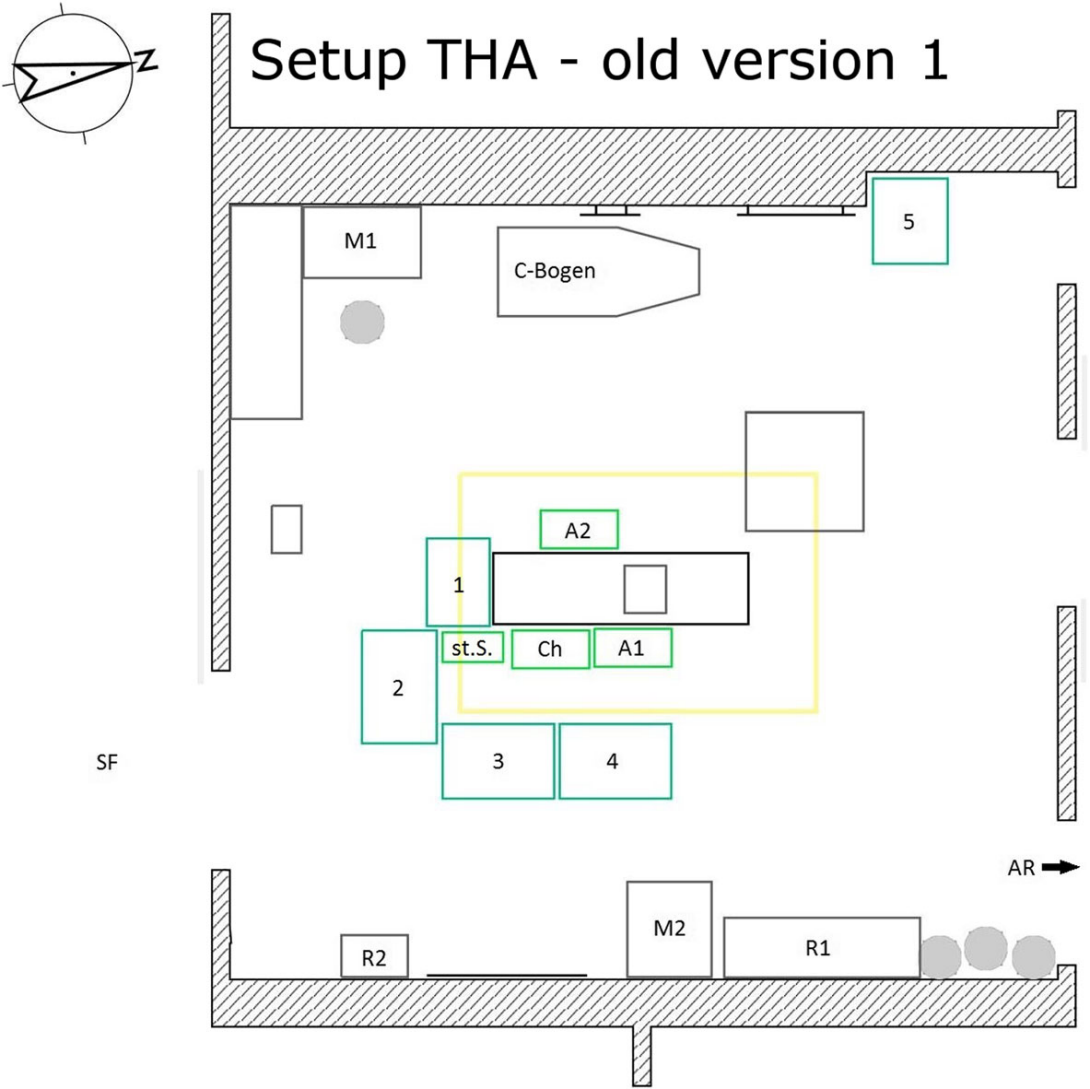
Setup THA – old version 1. Ch surgeon, St.S. scrub nurse, A1 the first assistant, A2 the second assistant, 1–4 instrument tables, M1/ M2 monitor 1/ monitor 2, R1/ R2 shelves 1/ shelves 2, SF sterile corridor, AR recovery room

**Fig. 2:**
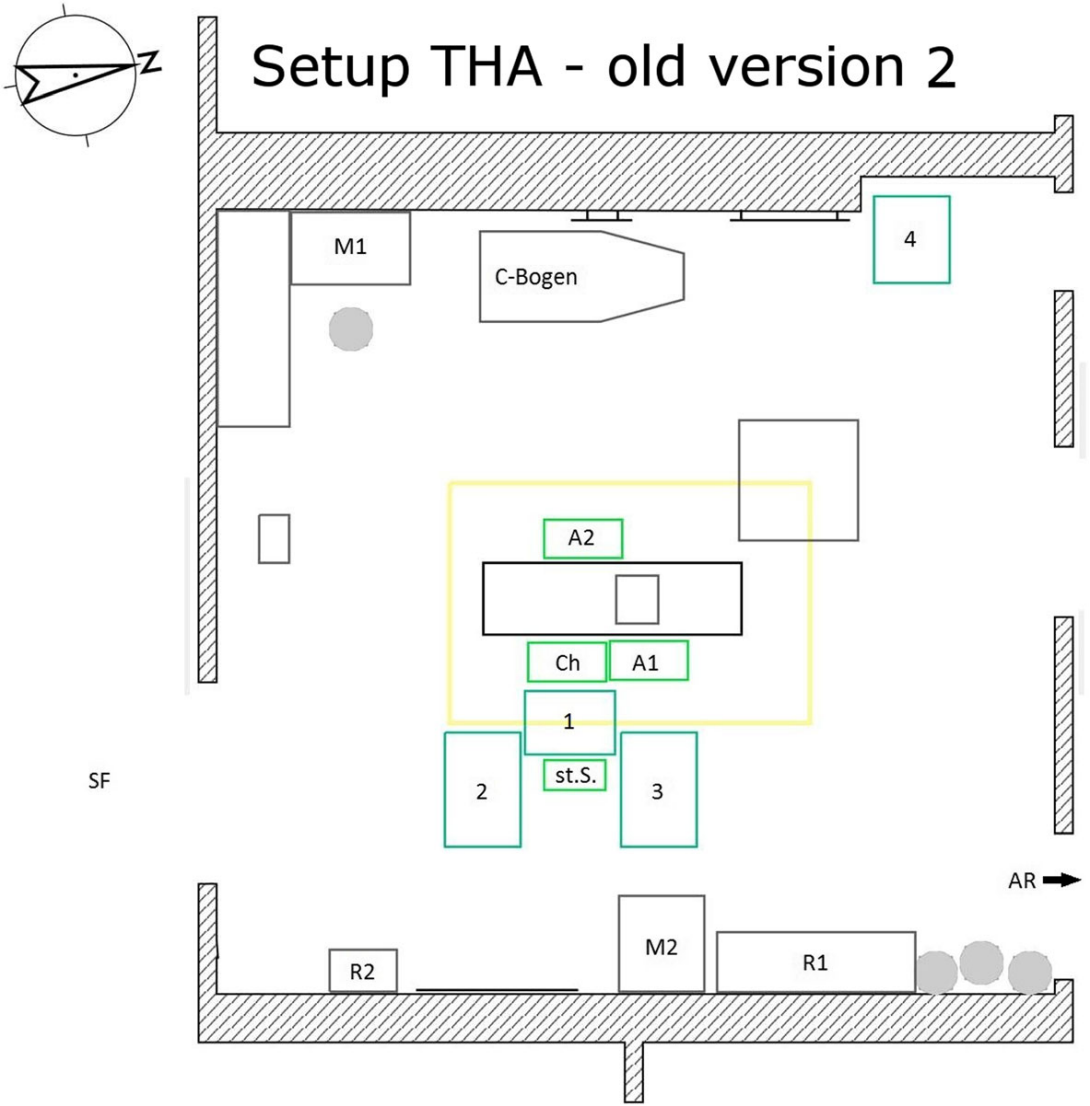
Setup THA – old version 2. Ch surgeon, St.S. scrub nurse, A1 the first assistant, A2 the second assistant, 1–4 instrument tables, M1/ M2 monitor 1/ monitor 2, R1/ R2 shelves 1/ shelves 2, SF sterile corridor, AR recovery room

Included in the study were elective primary hip and knee arthroplasties at the university maximum care hospital with maximum care endoprosthesis center in the period from April 2016 to December 2018. The surgeries were performed by five different surgeons (certified senior and main surgeons). To reduce the differences in surgical procedures between the surgeons, all surgeons performed operations in both the new and the old setup Figs. 1 and 2. Complex surgeries (dysplasia, fractures, posttraumatic conditions, revision surgery, etc.) and assisted surgeries, different implant systems and cementing procedures were excluded.

In the context of the underlying work, the existing OR setups for hip and knee arthroplasties were simulated and analysed with the computer program Delmia Quest [10] and new setup suggestions were developed based on these [9]. For the optimized setup, special attention was paid to the table position, optimization of times and improved practicability. The software systems and simulation models used for process optimization in the operating room and for planning and creating a surgery schedule have been validated by studies [2, 3, 5, 11, 12].

**Fig. 3:**
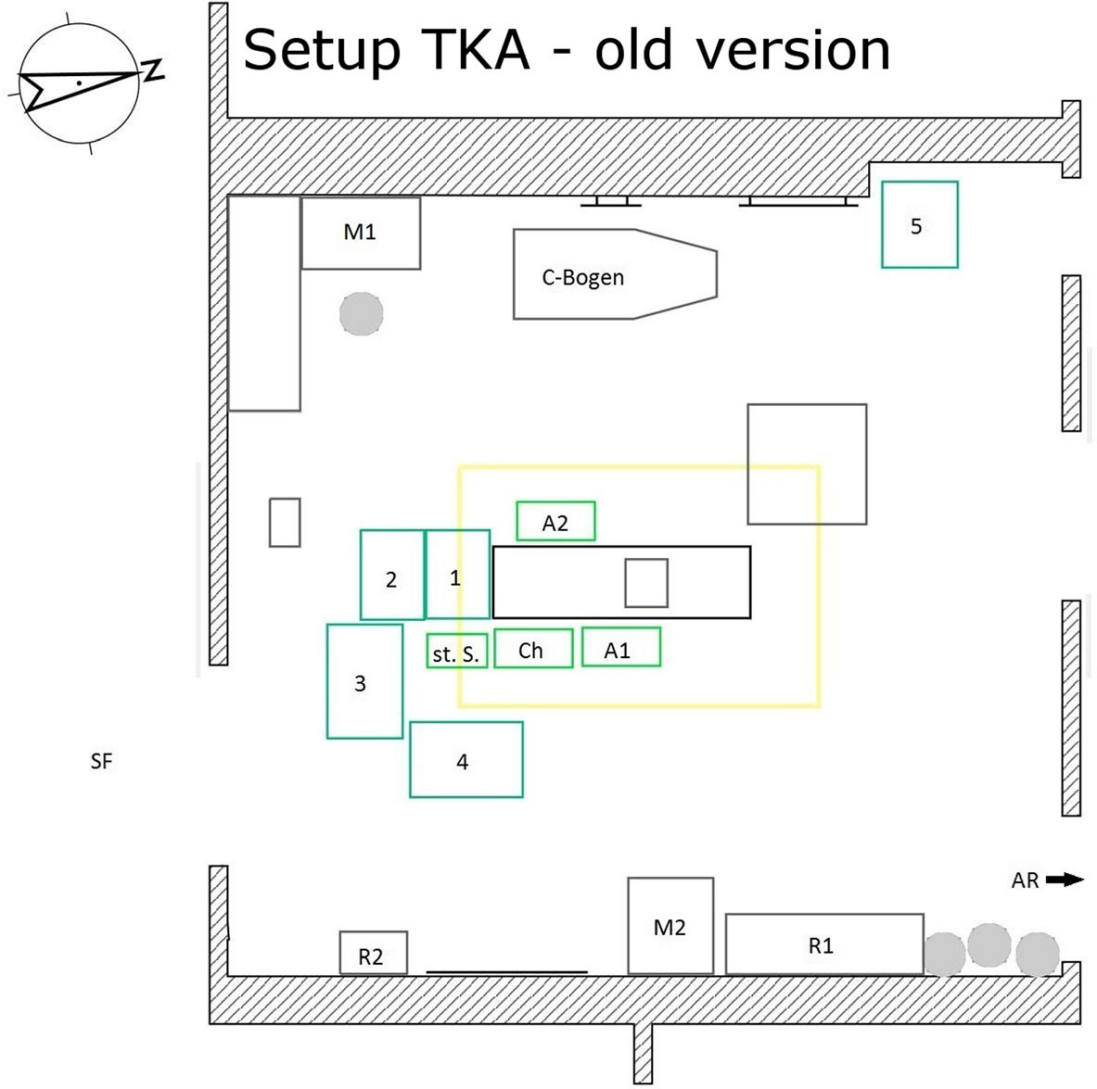
Setup TKA – old version. Ch surgeon, St.S. scrub nurse, A1 the first assistant, A2 the second assistant, 1–4 instrument tables, M1/ M2 monitor 1/ monitor 2, R1/ R2 shelves 1/ shelves 2, SF sterile corridor, AR recovery room

For hip arthroplasties, there were two different currently used setups and for knee arthroplasties, one setup was most frequently used. These were basically similar for both surgeries. On the one hand, there was the variant in which the scrub nurse was positioned directly behind the surgeon on the side to be operated on. The instrument tables were arranged in a U-shape around the nurse. The tables arranged in front were located between the nurse and the surgeon. On the other hand, there was a setup in which the nurse stood directly next to the surgeon and they were not separated by an instrument table. In this arrangement, the instrument table was positioned in a J-shape around nurse, surgeon and the first assistant. The tables were arranged from the foot end of the operating table to the back of the surgical team on the operation side (see Figs. 1, 2 and 3).

**Fig. 4:**
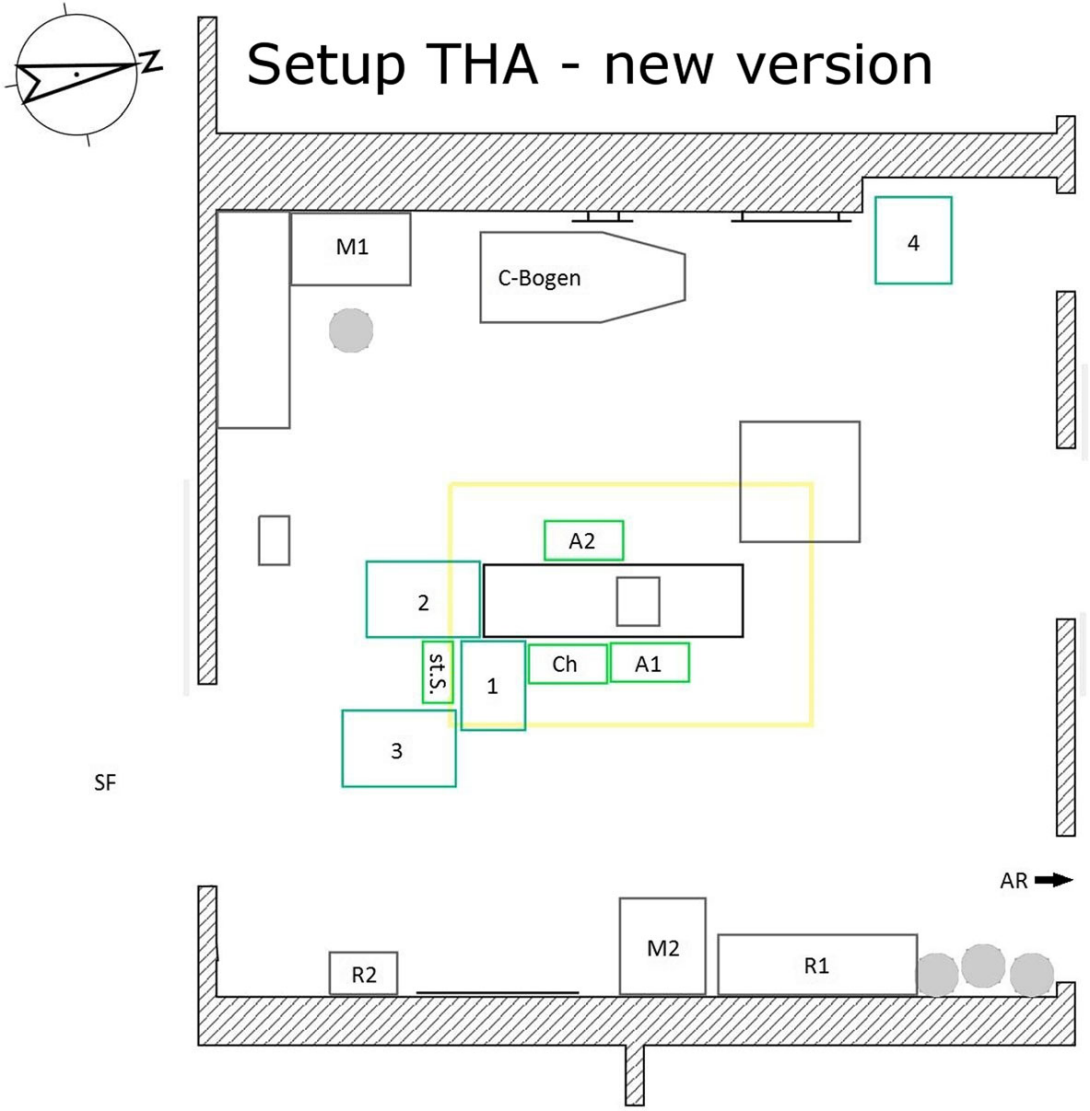
Setup THA – new version Ch surgeon, St.S. scrub nurse, A1 the first assistant, A2 the second assistant, 1–4 instrument tables, M1/ M2 monitor 1/ monitor 2, R1/ R2 shelves 1/ shelves 2, SF sterile corridor, AR recovery room

**Fig. 5:**
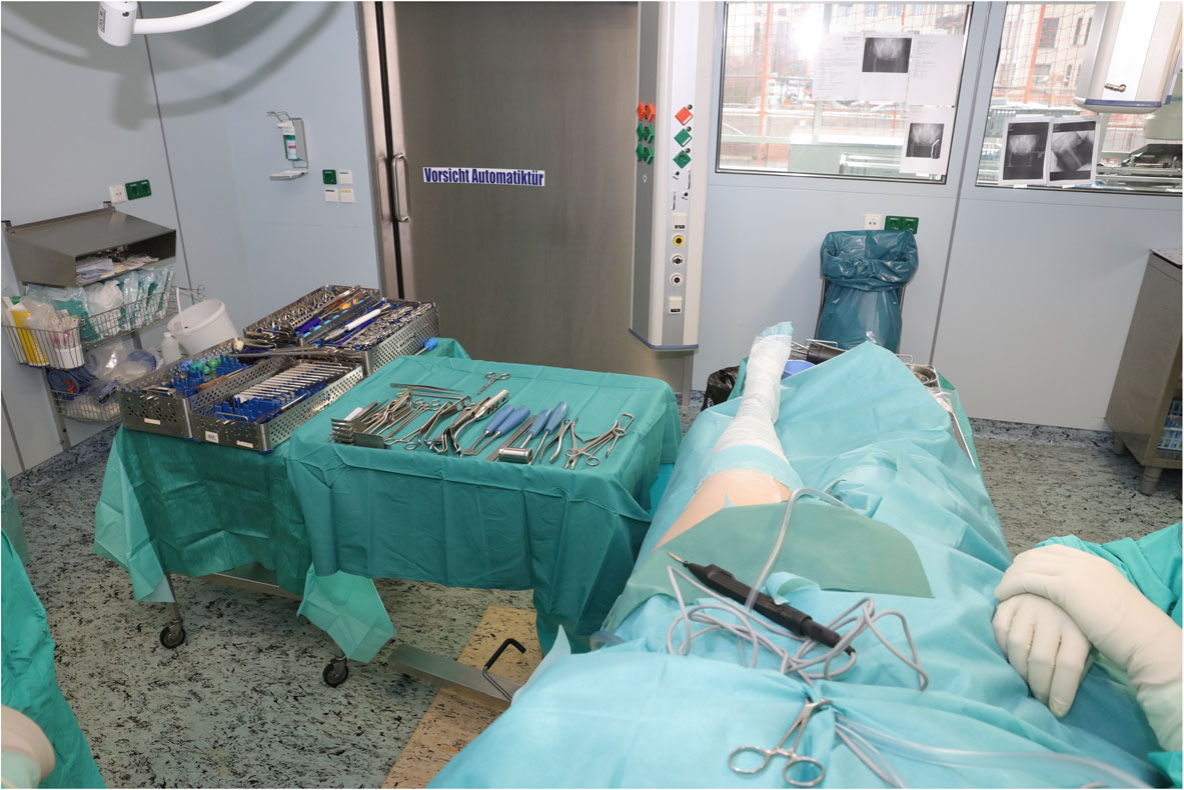
Setup TKA – new version Ch surgeon, St.S. scrub nurse, A1 the first assistant, A2 the second assistant, 1–4 instrument tables, M1/ M2 monitor 1/ monitor 2, R1/ R2 shelves 1/ shelves 2, SF sterile corridor, AR recovery room

Structure of the previously used setups (each for surgeries on the left side):

The optimized setup for the total hip arthroplasties consisted of an operating table and three instrument tables for the scrub nurse. The three instrument tables were arranged like a “U” around the nurse so that the nurse was oriented to the surgeon via the front table. For a hip endoprosthesis on the left side, the front table stood to the left of the operating table, the table to the left of the nurse stood directly behind the operating table and the table to the right of the nurse closed the “U” on the operation side. For an endoprosthesis on the right side, the table structure was mirrored. It was important that the rasps and instruments used to insert the shaft were placed on the outer table which was on the side of the leg to be operated on. The reason for this was that the surgeon had to change the position with the first assistant to prepare the shaft. The surgeon stood near the head end of the patient, further away from the scrub nurse. This required that the scrub nurse close this gap with the instrument tables. The nurse moved with the front and side tables behind the first assistant and therefore had the same handover distance to the surgeon as in the previous position (see Figs. 4 and 5).

**Fig. 6:**
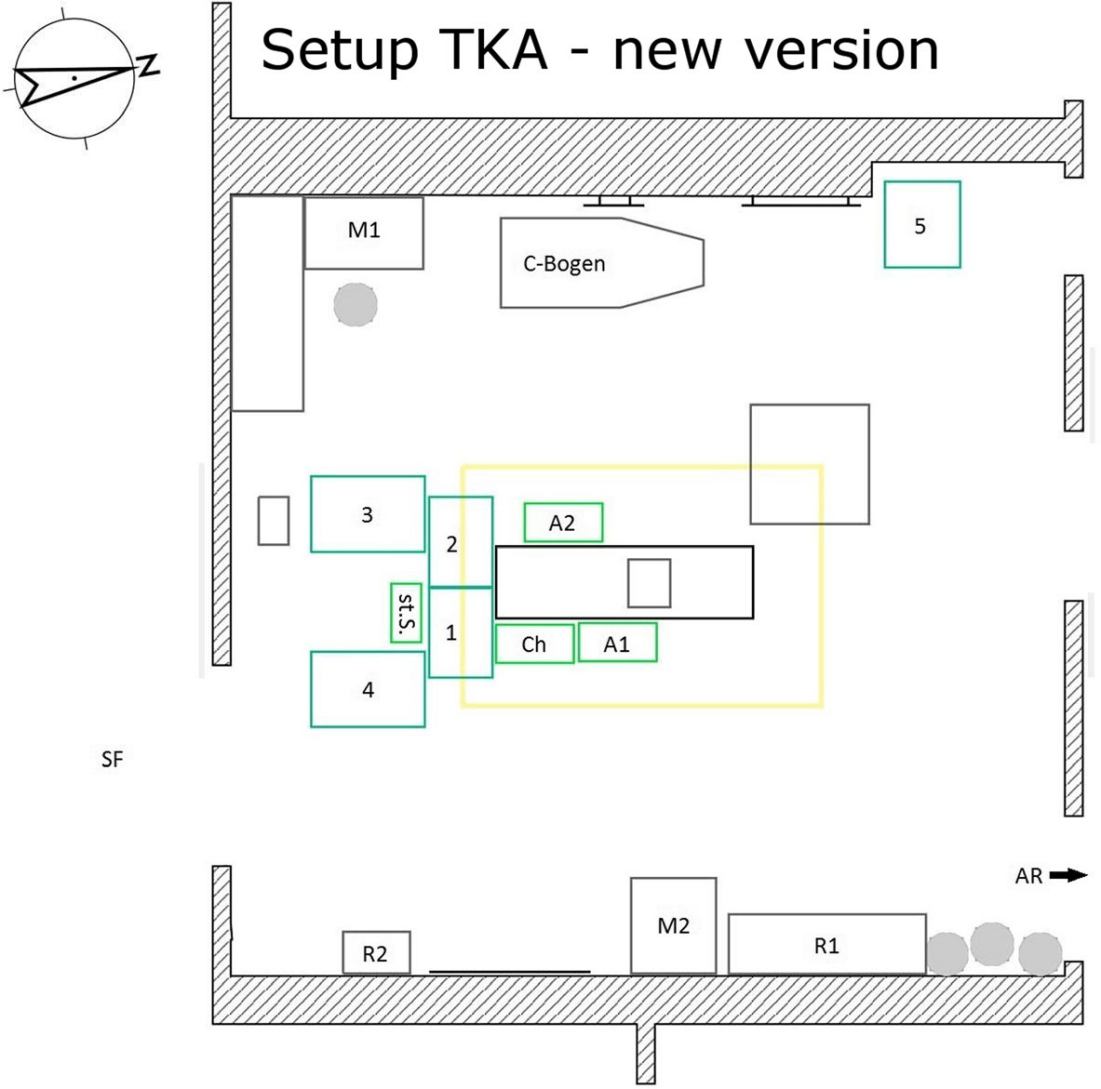
Structure of the new setup for HTEPs in the operating room

The new setup for the total knee arthroplasty was based on the total hip arthroplasty setup. Here it was possible to arrange the setup with three or four instrument tables. The structure of the “U” around the scrub nurse was retained. The scrub nurse stood directly behind the operating table and did not laterally move as in the setup for the hip. One table (or, if necessary, two tables if a total of four instrument tables were required) stood directly in front of the nurse and thus directly behind the operating table. The remaining two tables formed the “U” to the right and left of the nurse. The tables did not have to be moved during the entire knee endoprosthesis implantation (see Fig. 6).

Standardized observation sheets were used for the observation of the surgeries. The incision-suture time, which ranges from the incision to the end of the suture and the time between the incision, and suture beginning, which ranges from the incision to the beginning of the suture and thus represents the pure time for the orthopaedic surgical steps, were recorded. In addition, the handovers of the scrub nurse to the surgical team were recorded. Individual handover times were measured for each surgery, starting when the nurse touches the instrument until the surgeon holds it in his hand. In addition, the practicability of the setup from the outside was determined by observing the surgical procedure and consulting the surgical team. The focus here was on manageability, comfort, efficiency of instrument handovers, the handover distance and on the rotational movements that the staff had to do during the surgery to hand over instruments.

In addition, a questionnaire was developed to measure staff satisfaction with the new setup and to filter out possible setup difficulties. The questionnaire consisted of seven categories to assess the setup and was completed by the operating surgeon, the first assistant, the scrub nurse and the non-sterile nurse. The categories were rated on an ordinal scale from one point for “bad” up to five points for “very good”. The categories were manageability/efficiency, ergonomics/comfort, visibility, space, walking distance, hygiene and satisfaction. For each category a question was formulated. In addition, disadvantages, advantages and comments on the setup could be noted in free text.

The statistical data preparation and evaluation were done with the programs Microsoft Excel 2013 (Redmond, USA) and SPSS 24.0 (IBM, IL, USA). The metric scaled data were checked for normal distribution with the Kolmogorov-Smirnov test and then checked for differences with an independent Student’s *t*-test or alternatively with the non-parametric Mann-Whitney U test. The ordinal scaled questionnaire data were evaluated using the non-parametric Kruskal-Wallis test method. The significance level was set at the value 0.05.

## Results

For further evaluation, the two currently used setups for hip arthroplasties were combined as a baseline.

The data collection included 19 total hip arthroplasties in the currently-used setup and 15 total hip arthroplasties in the new setup as well as 9 total knee arthroplasties in the currently-used setup and 13 total knee arthroplasties in the new setup. These were recorded in each case in the period from April 2016 to October 2016 as initial data collection for the underlying study and in the period from September 2018 to December 2018 at the examination hospital. There were no differences in blood loss or other outcome parameters of patients between the two groups.

The patients were positioned in supine position and access was from lateral according to Bauer (THA) or parapatellar medial (THA). The hips were operated in the non-cemented system Mathys (CBC Evolution or Optimys, cup Aexys) and the knee in the cemented system according to the DePuy Synthes Companies (LCS Complete). A blood arrest system at the knee was only used for cementing.

In the group of primary hip arthroplasties, the incision-suture-beginning-time, the handovers/minute and the pure handover times were compared against each other. For both setup variants, handover times were measured, starting when the nurse touched the instrument until the surgeon held it in his hand. All surgeries included, 90 times were recorded in the currently-used setup and 94 times in the new setup. The average value was 1.82 +/− 1.43 s and the median 1.41 s in the currently-used setup. For the new setup, the average value was 1.08 +/− 0.78 s and the median 0.92 s. Thus, the handover times in the group of hip arthroplasties differed highly significantly (*p* < 0.001).

**Fig. 7:**
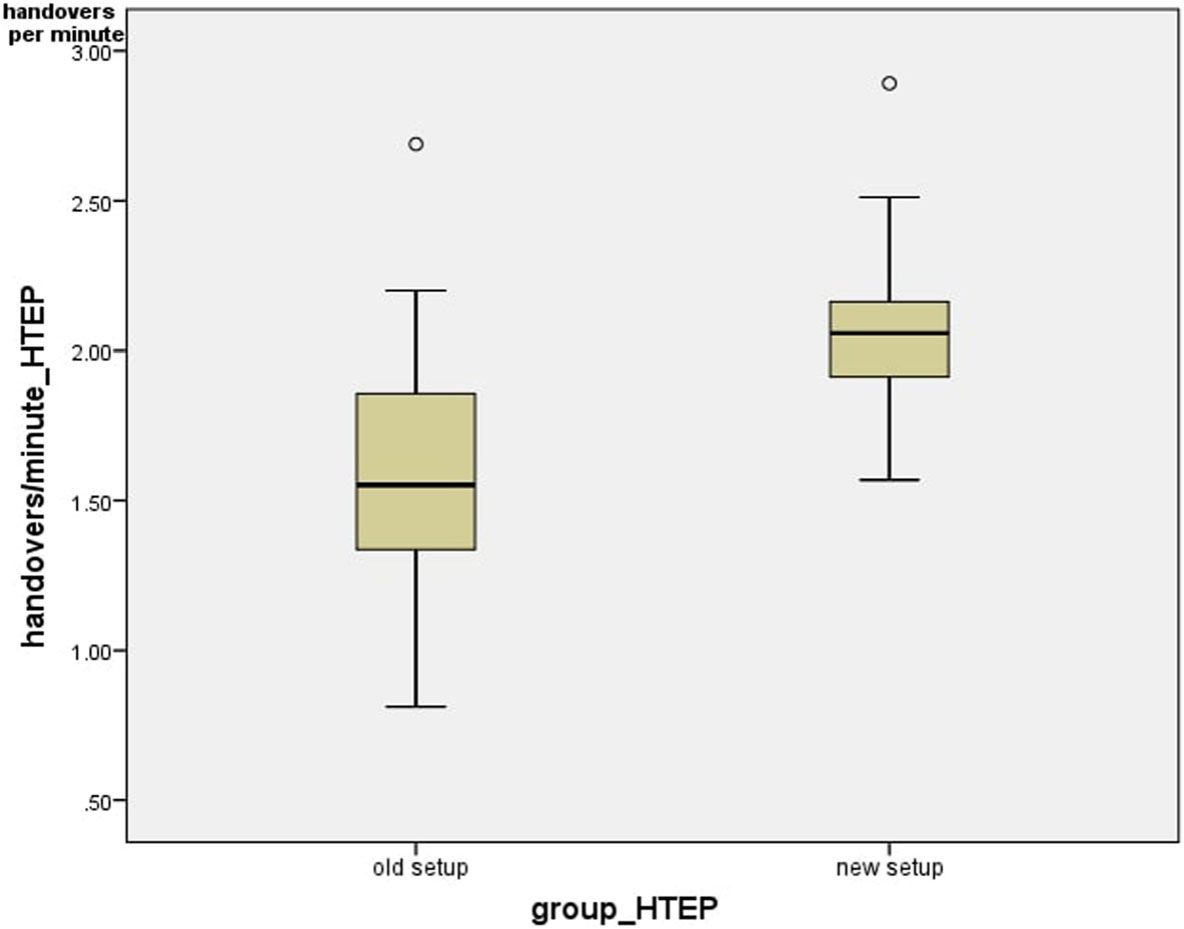
Time between incision and suture beginning_HTEP

**Fig. 8:**
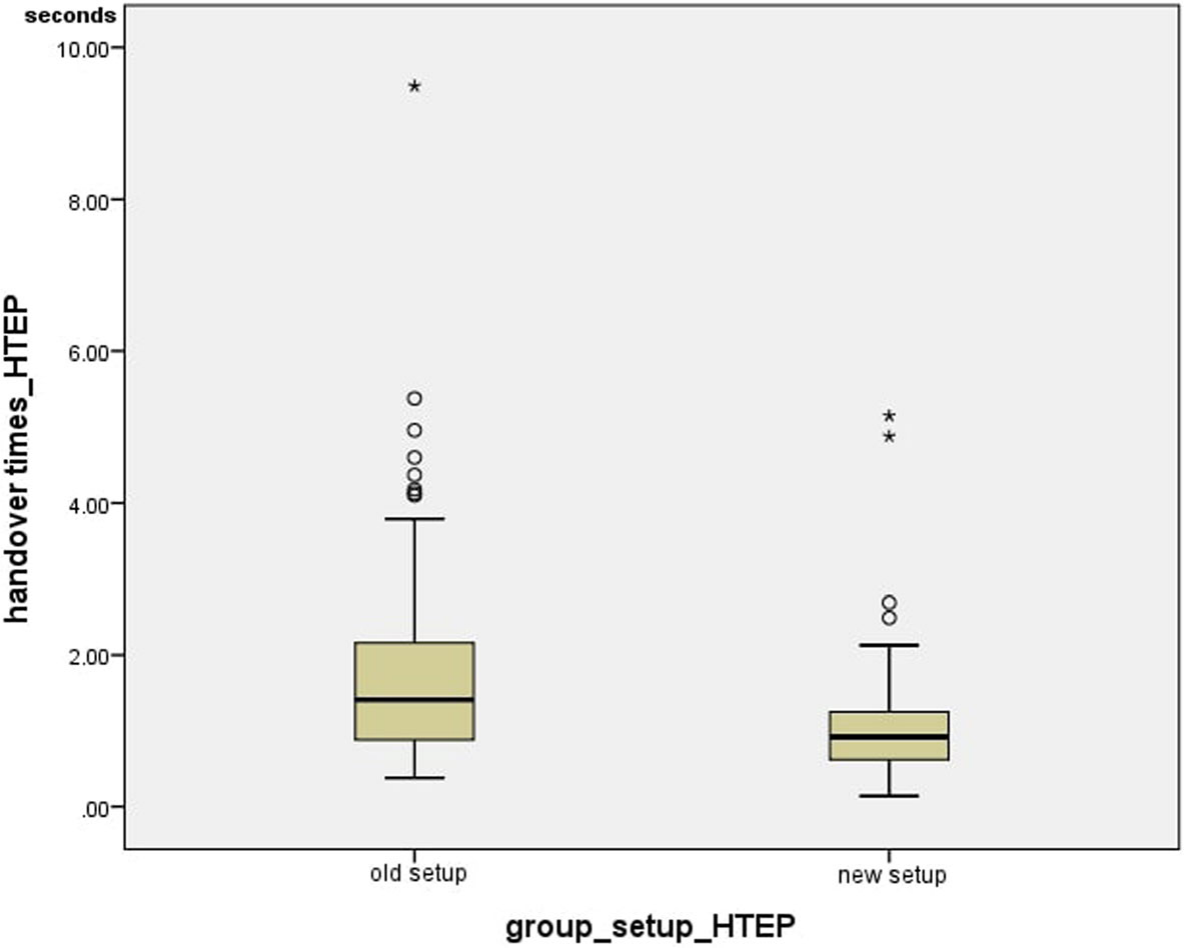
Handovers per minute_HTEP

Looking at the handovers that could be made per minute in the respective setups, the average value was 1.62 +/− 0.45 handovers per minute in the previous setup and 2.10 +/− 0.32 handovers per minute in the new setup. There was a significant difference (*p* = 0.001) in the number of handovers made per minute in the group of hip arthroplasties (Figs. 7 and 8).

**Fig. 9:**
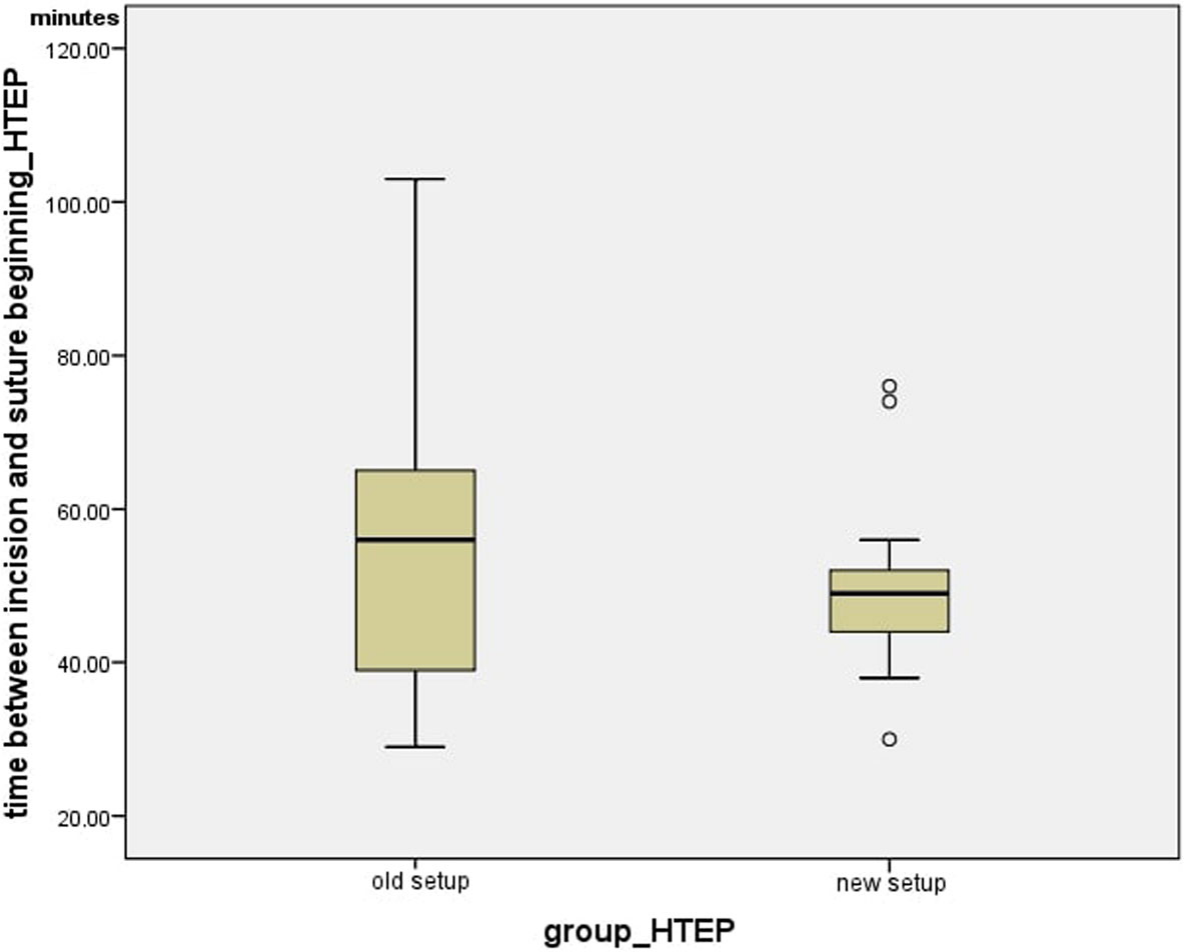
Handover times_HTEP

The average time between incision and suture beginning was 53.89 +/− 18.92 min in the currently-used setup and 49.733 +/− 12.18 min in the new setup, while the median was 56.00 min for the previous setup and 49.00 min for the new setup. The time between incision and suture beginning for the currently-used and the new setup in the group of hip arthroplasties did not differ significantly (*p* = 0.466) Fig. 9.

There was no significant difference in the operated side between the two setups used (Old setup 9/19 right 47%; new setup 8/15 53%; *p =* 0,73). Also in Body Mass Indey (BMI) there were no significant differences between the groups. (old setup median BMI: 25 (17–36); new setup median BMI: 27.5 (19–32); *p =* 0,819).

In the group of the total knee arthroplasties, the same values for the total hip arthroplasties were recorded and evaluated.

**Fig. 10:**
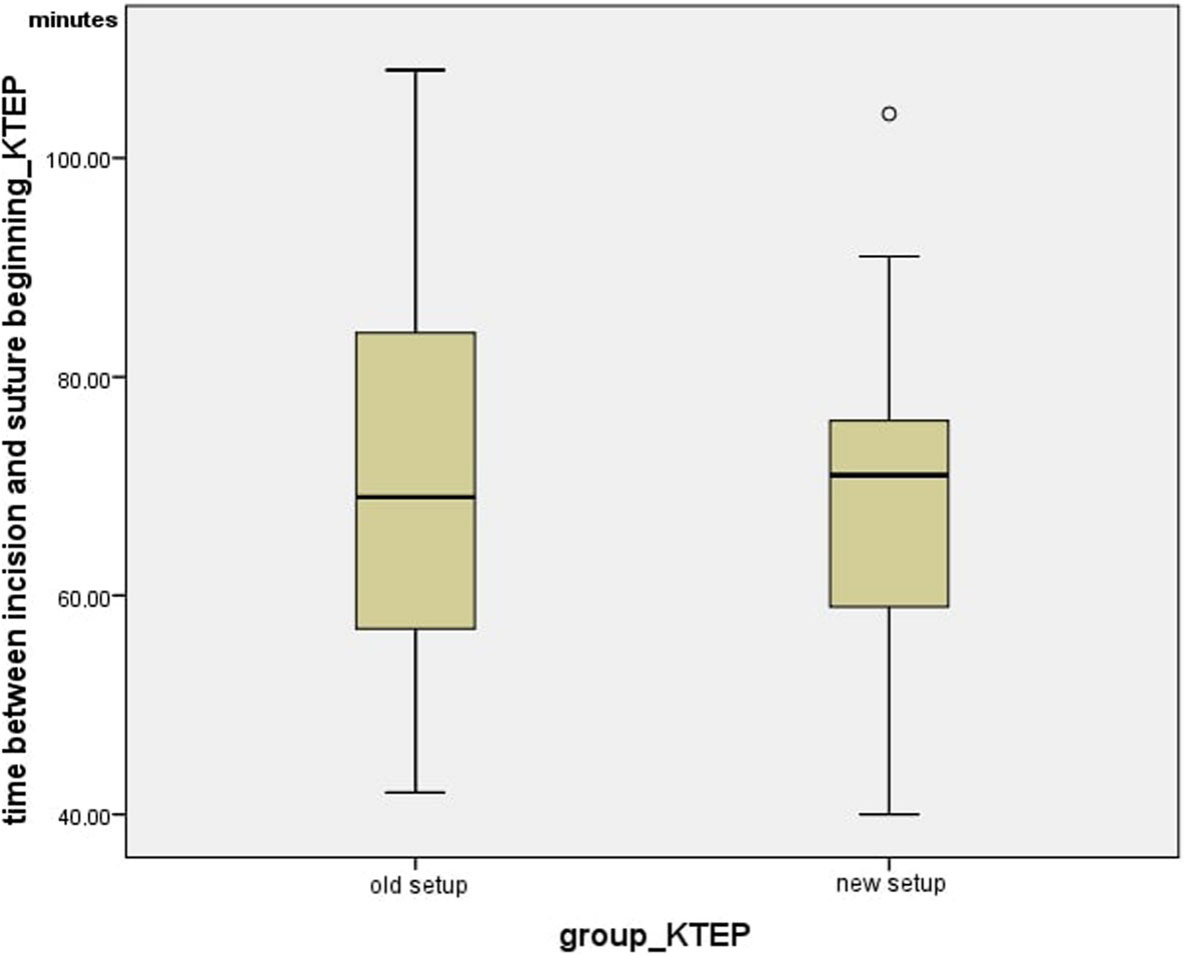
Time between incision and suture beginning_KTEP

Also, for the total knee arthroplasties, the number of handovers per minute was determined in the different setups. In the currently-used setup, the average value was 1.83 +/− 0.38 and the median was 1.75 handovers/minute. In the new setup, the average value was 2.40 +/− 0.35 and the median was 2.34 handovers/minute. This means that the number of handovers/minute differed significantly (*p =* 0.002) (Fig. 10).

**Fig. 11:**
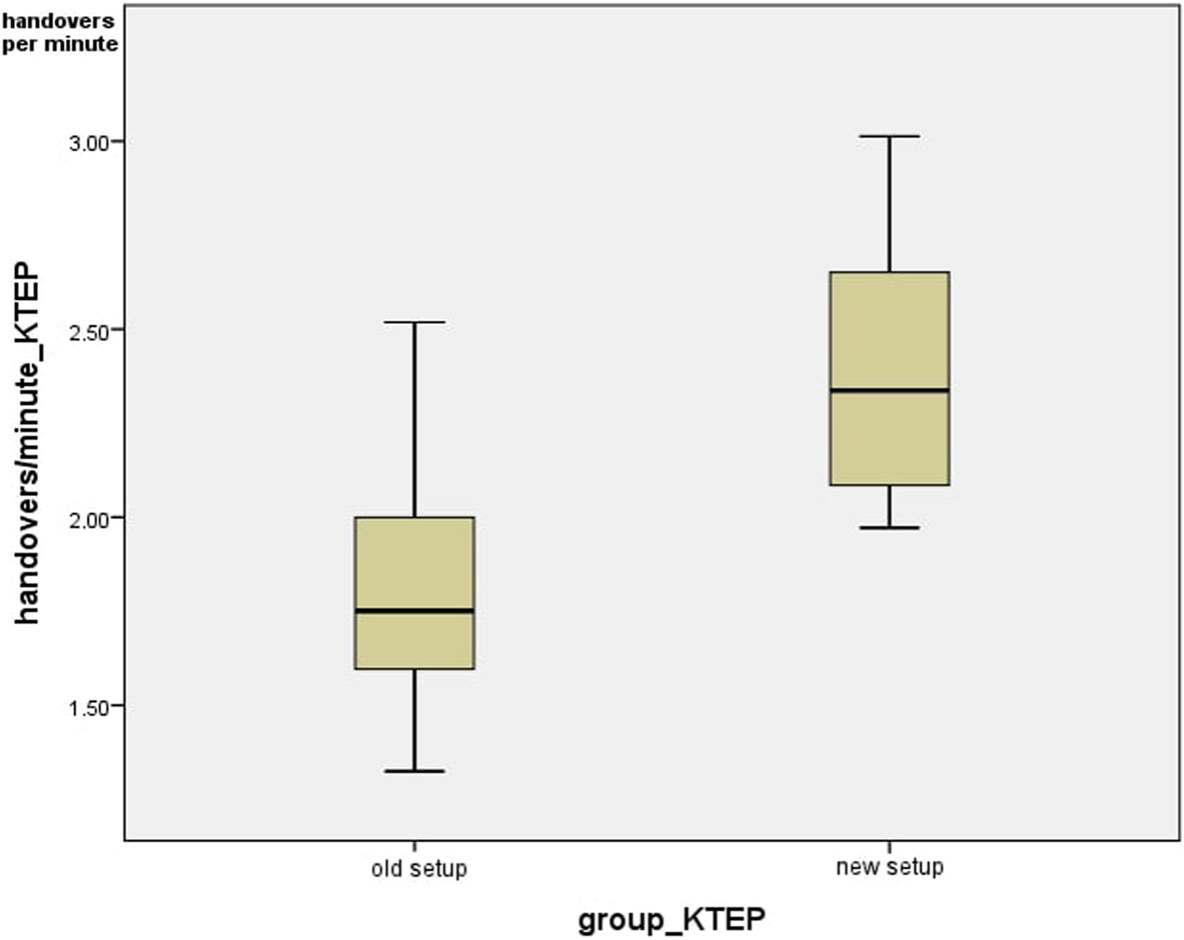
Handovers per minute_KTEP

The handover times for the knee arthroplasties were measured exactly like those for the hip arthroplasties: 43 times were recorded in the currently used and 134 times in the new setup. The average value in the previous setup was 1.06 +/− 0.64 s and in the new setup 0.91 +/− 0.59 s, while the median was 0.88 for the previous setup and 0.77 for the new setup. The handover times did not differ significantly (*p =* 0.152) (Fig. 11).

**Fig. 12:**
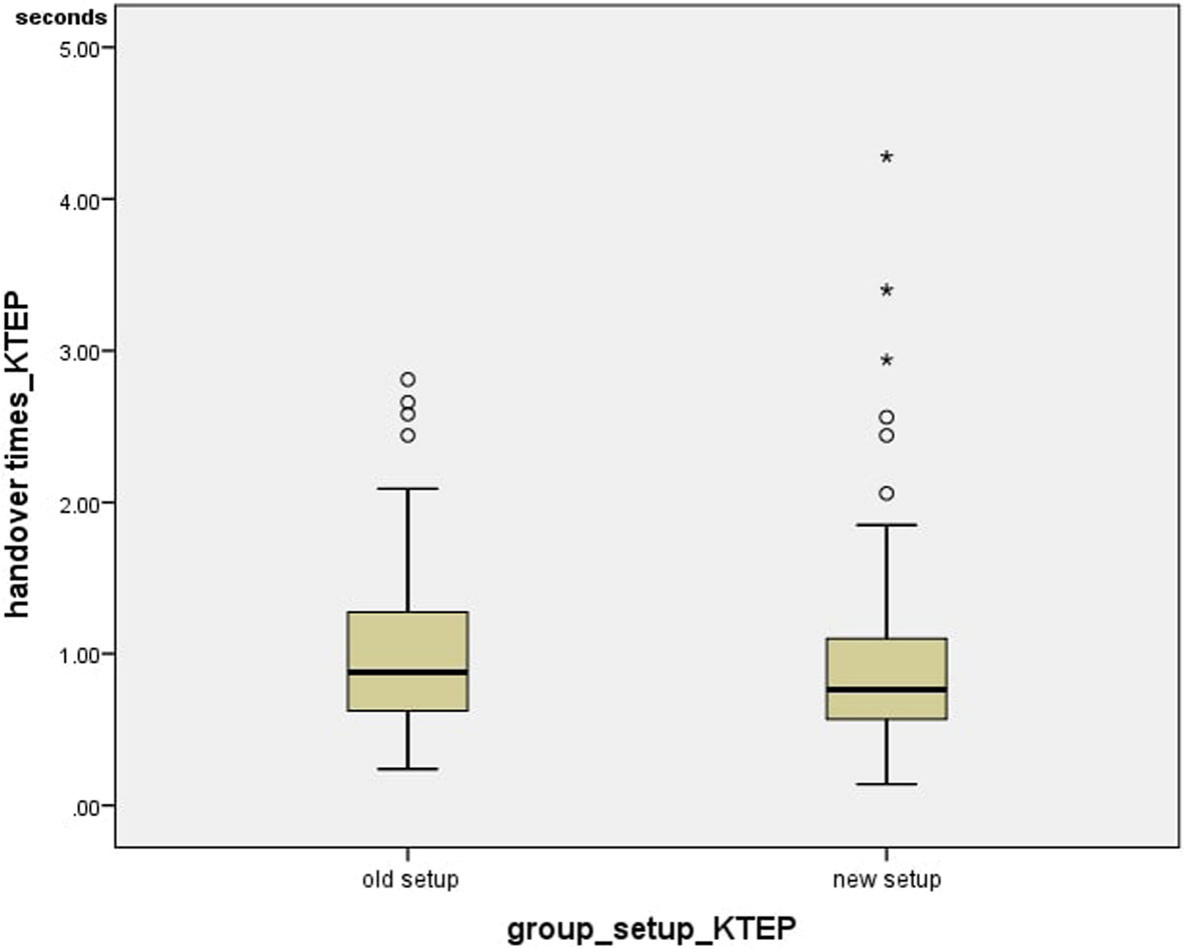
Handover times_KTEP

The average value for the time between incision and suture beginning in the currently-used setup was 71.11 +/− 20.72 min and the median was 69.00 min, while the average time in the new setup was 70.69 +/− 17.12 min and the median was 71.00 min. This means the time between incision and suture beginning for the setups for knee arthroplasties did not differ significantly (*p* = 0.959) (Fig. 12).

There was no significant difference in the operated side between the two setups used. (Old setup 3/9 right 33%; new setup 7/13 53%; *p =* 0.45) Also in Body Mass Indey (BMI) there were no significant differences between the groups. (old setup median BMI: 27 (20–34); new setup median BMI: 28 (21–34); *p =* 0,56).

The results of the questionnaire showed a very high level of staff satisfaction with the new setup for both hip and knee arthroplasties. (Table 1) Most of the median values were between 4 and 5 points and never fell below 3. Especially the surgeon seemed to be particularly satisfied. For the hip arthroplasties, he awarded the median for all categories except manageability/efficiency (median 4.5) and walking distance (median 4) with the full score of 5 points. For the new setup for total knee arthroplasties, the surgeon even gave the highest score (median 5) in all seven categories.

**Table 1:** Median points divided into the various occupational groups

hip	surgeon	scrub nurse	non-sterile nurse	the first assistant
**completed questionnaires**	10	10	7	7
**manageability/ efficiency**	4.5	4	4	5
**ergonomics/ comfort**	5	5	4	4
**visibility**	5	3	4	4
**available space**	5	5	4	5
**walking distance (non-sterile nurse)**	4	5	3	4.5
**hygiene**	5	4.5	5	5
**satisfaction**	5	4	4	5
**knee**	**surgeon**	**scrub nurse**	**non-sterile nurse**	**first assistant**
**completed questionnaires**	9	6	8	7
**manageability/ efficiency**	5	4	4	4
**ergonomics/ comfort**	5	4.5	4	5
**visibility**	5	5	5	5
**available space**	5	4.75	4	5
**walking distance (non-sterile nurse)**	5	3.5	3	4.5
**hygiene**	5	4	4	5
**satisfaction**	5	4.5	4	5

The Kruskal-Wallis test showed that for hip arthroplasties only the category “visibility” differed significantly in the four occupational groups. (Table 2).

**Table 2:** Kruskal-Wallis-Test

variable / question items	***p***-value (knee)	***p***-value (hip)
manageability	0.022	0.126
ergonomics	0.017	0.243
visibility	0.261	*P* < 0,001
available space	0.003	0.356
Walking distance	0.004	0.197
hygiene	0.009	0.73
satisfaction	0.001	0.42

- questionnaire: comparison of 4 occupational groups (surgeon, scrub nurse, non-sterile nurse, the first assistant)

- statistical test procedure: use of non-parametric Kruskal-Wallis test

- marked yellow: The difference between the 4 groups was significant (*p* < 0.05)

- data: ordinal scale level, i.e., indicate only medians

For knee arthroplasties, all question items except for “visibility” differed significantly according to the Kruskal-Wallis-Test.

In the columns for free text annotations, above all the structural conditions in the operating room were noted, which restrict space and obstruct walking routes. Some scrub nurses mentioned the unergonomic way of reaching the surgeon forward over the instrument table.

## Discussion

The optimization of the OR setups is very important for increasing the efficiency as well as for quality assurance. Especially, in the case of very frequently performed surgeries, such as hip and knee arthroplasties, the quality in the operating room should be checked repeatedly and potential for improvement should be optimally exploited [7].

The results for the total hip arthroplasties showed an increase in efficiency by optimizing the operating room setups. A highly significant (*p* < 0.001) difference in handover times, a significant (*p* = 0.001) difference in the handovers per minute as well as a tendency towards a reduction in the time between incision and suture beginning could be determined, even if this was not statistically significant (*p* = 0.466).

This study showed that the work steps that are directly related to the table position, such as handing over surgical instruments, can be performed much more quickly and uncomplicatedly, thus optimizing the entire surgical procedure. Although the time between incision and suture beginning did not differ significantly, we assume that the reason for this is the training period with a training curve into the new setup and low number of cases. To reduce distortion due to interindividual deviations (different operators and scrub nurses), it was determined how many handovers were made per minute, since this value is more valid than the pure time between incision and suture beginning, which is influenced by many other factors. The significant difference in the number of handovers per minute (*p* = 0.001) shows the notable increase of efficiency. This was also demonstrated by the highly significantly (*p* < 0.001) faster handover times in the new setup.

In the new setup, there are more handovers per minute, but generally also more handovers in total than in the old setup. We noticed this aspect when evaluating the data and the higher number of handovers could cause an increase in handovers per minute. Unfortunately, we couldn’t find an exact reason for this phenomenon even after consultation with the different surgeons. We could imagine interindividual differences between the surgeons or that the new setup invites more handovers due to the easier passing of the instruments and the ergonomics.

To ensure that the surgical procedure for hip arthroplasties functions as optimally as possible, it is important to ensure that the table position is changed according to the description in the Materials and methods part when the surgeon and the first assistant change positions for inserting the shaft. The scrub nurse moves the front and side tables behind the first assistant, with the rasps and instruments used for shaft insertion lying on the outer table on the side of the leg to be operated on. But if the rasps for the insertion of the shaft are on the table, which is directly behind the operating table and does not have to be moved, the way for the scrub nurse becomes too long, why it is important to ensure at the beginning of the surgery that the instrument table for the shaft is on the side to be operated on.

Looking at the results for total knee arthroplasties, the increase of efficiency was initially not as visible as for hip surgeries.

The handover times per minute differed significantly (*p* = 0.002), the handover times did not differ significantly (*p* = 0.152) and the time between incision and suture beginning did not differ significantly (*p* = 0.959). As with the hip arthroplasties, we assume that this was due to the training period with a training curve in the new setup and the low number of cases. On the other hand, the surgical procedure for knee operations is very complex. The insertion of a knee endoprosthesis requires very large surgical steps during which there are hardly any handovers, but there are again phases with very many handovers [13]. These phases with large surgical steps are independent of the table position, more susceptible to interindividual differences between the surgeons and constitute a large part of the surgery time. They are, to a great extent, influenced by the surgeon’s performance, complications and other factors. Nevertheless, there were also significant differences in the handovers per minute (*p =* 0.002), which could be assumed to be a valid measure of efficiency.

A review by Pokrywka and Byers on airflow and contamination of surgical wounds described that unnecessary movement during the surgery, as well as entering and leaving the room, interrupts the sterile airflow and contaminants are not sufficiently removed from the sterile area. With a high level of activity in the operating room, the bacterial count of microorganisms in the air increases [14]. Contamination of the air in the immediate surgical area can also result in contamination of the surgical field, since the air plays a major role in the transmission of pathogens during the surgery [15]. In the new surgical setup, special attention was therefore paid to optimizing hygiene standards. Observation of the operating activities showed that the personnel, especially the surgeon and the scrub nurse need to carry out considerably fewer rotational movements in order to hand over the instruments. Unfortunately, this could not be measured validly and therefore referred to pure observation. Theoretically, the nurse only has to rotate 90 degrees to the instrument table on her right and left side to reach the instruments. However, the observations showed that the nurse needed to rotate considerably less by using the freedom of movement of the arms. The surgeon, as well, has a theoretical rotational movement range of 90 degrees to the scrub nurse, which he also reduces by using the arms. In the currently-used setup, significantly more rotational movements are necessary [9], so that air turbulence could be reduced with the new setup. In addition, it is not necessary to hand over the instruments from behind via the surgeon’s back, as was often the case with the currently-used setup. If the instruments were handed over via the back, the surgeon could not ensure that all instruments were visibly clean [16] and the risk for contamination would therefore increase.

In the new setup, the instrument tables are hypothetically closer to the operating table and thus more in the protected area, which is characterized by a stable flow of air filtered from suspended matter, which is virtually sterile and separates the area of operating table and instrument table from the rest of the surroundings [15].

The results of the questionnaire showed a very high level of staff satisfaction with the new setup for both hip and knee arthroplasties.

In the setup for hip arthroplasties, only the item “visibility” differed significantly between the four occupational groups. In the case of knee arthroplasties, all items except “visibility” differed significantly. This could be due to the fact that everyone involved in knee surgery was close to the joint to be operated on than during hip replacement surgery and therefore all had a very good view. The setup for the knee enabled the scrub nurse to stand directly at the end of the table and thus gave a very good overview. In the case of hip arthroplasties, however, the nurse was positioned laterally offset from the operating table to be able to reach the surgeon easily, but thus lost some visibility, because in the case of the large surgical steps, one of the surgeons often stood between the nurse and thereby clear view of the operating field. The surgeon also stood directly at the hip and had an optimal view. Since the instrument tables for knee arthroplasties were located behind the operating table, they took up more space in the room and thus had a very strong influence on the walking distance, the available space for each individual as well as compliance with hygiene standards and overall satisfaction. As a result, although everyone could see well, all the other categories differed significantly between occupational groups. The setup for the hip arthroplasties was more at the side of the operating table and this tood up less space in the room, or rather fit better into the operating room. Therefore, all categories except “visibility” were assessed similarly by all occupational groups and did not differ significantly.

The questionnaire showed a very high level of satisfaction, which is particularly important because the satisfaction of the individual team members and performance are positively correlated [17]. This shows that it is important to look at the staff satisfaction during every intervention, because only satisfied team members can achieve full performance. The publication discussed the performance-caused satisfaction theory, but ultimately assumed a circular relationship [17].

Further possibilities for increasing efficiency were discussed in Fong’s publication. Among other things, it mentioned cooperation always with the same team, less use of instruments by the surgeon and increased familiarity within the team. It is also emphasized that communication controls the operating room and teamwork is the foundation for a successful surgery. Every team member should be heard [1]. From these points of view, the importance of the questionnaire for the good execution of the new setups becomes apparent. If all team members are involved and are satisfied with the new setup, it can be implemented even better. Therefore, it is even more important that the results of the survey are so positive and that all team members report a high level of satisfaction with the setup for both hip and knee arthroplasties.

### Limitations

Due to the small and inconsistent number of cases, no change in surgery time could be detected. Even assuming a learning curve with the new setup, larger case numbers would be necessary to confirm this assumption. Therefore, no conclusive statement on the reduction of surgery time was possible in this study. Individual procedures of each surgeon contributed to the contamination of the data. This was corrected as all surgeons performed operations in both the old and the new setup.

## Conclusion

All in all, it can be said that the new setup is quite a practical alternative for both hip and knee arthroplasties. It optimizes the events in the operating room in many ways. It offers saving of time and increases efficiency by allowing more handovers per minute. Also, it supports the comfort and satisfaction of the surgical staff. It ensures that instruments can be handed over efficiently. We expect to improve the implementation of hygiene standards by reducing the rotational movements and ensuring the permanent monitoring of the sterile area. The suggested table positioning depends on the individual circumstances of the different clinics. It should be considered as a suggestion but not as an absolute recommendation.

## Data Availability

The datasets used and/or analyzed during this study are available from the corresponding author upon reasonable request.

## Abbreviations

A1: First assistant
A2: Second assistant
AR: Recovery room
AE: German Society for Endoprosthetics
BVOU: Professional Association of Specialists for Orthopaedics and Trauma Surgery
BMI: Body Mass Indey
Ch: Surgeon
DGOOC: German Association for Orthopaedics and Orthopaedic Surgery
M1/ M2: Monitor 1/ monitor 2
R1/ R2: Shelves 1/ shelves 2
OR: Operating Room
SF: Sterile corridor
St.S.: Scrub nurse
THA: Total Hip Arthroplasty
TKA: Total Knee Arthroplasty
1–4: Instrument tables
